# Death and Rebirth of the Thalidomide Molecule: A Case of Thalidomide-Induced Sensory Neuropathy

**DOI:** 10.7759/cureus.13140

**Published:** 2021-02-04

**Authors:** Hassan Kesserwani

**Affiliations:** 1 Neurology, Flowers Medical Group, Dothan, USA

**Keywords:** molecular pharmacology, painful neuropathy, toxic neuropathies

## Abstract

The thalidomide molecule is a remarkable molecule that exists in a racemic mixture of optical isomers. In the 1950s, due to its teratogenicity, the levorotatory isomer led to its dramatic downfall. However, the molecule with its panoramic mechanisms of action and its uncanny ability to intercalate within the geometry of deoxyribonucleic acid (DNA), led to its remarkable renaissance; thalidomide being United States Food and Drug Administration (FDA)-approved for at least 13 different indications ranging from multiple myeloma to leprosy to glioblastoma. Thalidomide-induced polyneuropathy is usually reversible and is the rate-limiting step in its long-term use. The development of a polyneuropathy is invariably associated with a cumulative dose exceeding 20 grams. However, the polyneuropathy is almost always a sensory neuropathy. Asymmetry, bona fide weakness such as difficulty standing on the heels, a poly-ganglioneuropathy pattern with widespread or patchy numbness and sensory ataxia should raise a red flag and an alternative diagnosis should be considered. We present a typical case of a thalidomide-induced sensory neuropathy in order to highlight the resurgence of thalidomide use in clinical practice. We review the literature and outline the molecular biology of the thalidomide molecule.

## Introduction

Thalidomide is a versatile molecule. It is a derivative of glutamic acid and it has a panorama of actions that includes specific and broad antiinflammatory, immune-modulatory actions and inhibition of angiogenesis. On the downside, one of its chiral enantiomers has notoriously been associated with teratogenesis. Thalidomide is a racemic mixture of two enantiomers rectus R- and sinister S- optical isomers. They are readily interchangeable, the R-isomer is a sedative and the S-isomer is a teratogen [1].

As an analogue of glutethimide, it was marketed as a sedative, and as an anti-emetic for morning sickness with catastrophic results. Thalidomide embryopathy was an epidemic of congenital malformations between the years 1957 and 1961. Early exposure during pregnancy, between days 20 and 36 after fertilization, led to ocular, auditory, upper and lower limb and cranial nerve (facial motor nerve) malformations. Autism, epilepsy, and urogenital defects were also reported. Only one-half of the infants exposed to thalidomide survived [2]. 

Thalidomide's pleomorphic effects on the immune system, anti-proliferative effects and its anti-angiogenesis properties have led to its spectacular renaissance. It is now a United States Food and Drug Administration (FDA)-approved drug for a bewildering variety of at least 13 different diseases or disease states including erythema nodosum leprosum, multiple myeloma, acquired immune deficiency syndrome (AIDS)-wasting syndrome, recurrent aphthous ulcers and stomatitis in the immuno-suppressed, graft-versus-host disease, AIDS-related Kaposi's sarcoma, primary malignant glioma, myelodysplastic syndrome, various systemic lupus erythematosis states, Behcet's syndrome, prurigo, refractory Crohn's disease, and peripheral thymic (T)-cell lymphoma [3]. 

The polyneuropathy induced by thalidomide is mostly axonal and rarely demyelinating. It is typically a sensory neuropathy and is rarely a sensory-motor polyneuropathy. When sensory, the large fibers (touch, pressure, proprioception) are more affected than the small fibers (pain, temperature) [4]. Serum from patients treated with thalidomide for prurigo and injected into cultured rabbit dorsal root ganglia was associated with a neuronopathy (disease of the dorsal root ganglia) [5]. The cereblon (CRBN) gene codes for the enzyme (E)-3 protein-ligase-complex that mediates the ubiquitination and proteasomal degradation of proteins. This is required as a clearing mechanism of proteins during limb budding and sculpting during embryogenesis. In a study of 82 patients treated with thalidomide for multiple myeloma, carriers of the CRBN cytosine-cytosine (CC)-genotype had a 14-fold higher risk of a polyneuropathy [6].

## Case presentation

We present the case of a 64-year-old woman with bone marrow biopsy-proven (30% plasma cells) stage-III immunoglobulin (IgG) lambda multiple myeloma. She had a corresponding anemia but no hypercalcemia or lytic bone lesions. Remission was achieved with a course of decadron, cyclophosphamide and melphalan. Maintenance therapy was with oral thalidomide was done for three years after which she relapsed and developed osteonecrosis of the right maxilla, which was successfully treated with radiotherapy. Remission was subsequently achieved with oral lenalidomide.

One year after starting treatment with thalidomide, initially at 50 milligrams (mg) daily and slowly escalated up to 200 mg daily, she developed numbness, tingling and burning pain of the feet. The neuropathic symptoms were successfully treated with pregabalin at a dose of 150 mg twice daily. After two years of symptomatic treatment with pregabalin for the neuropathic pain, she was successfully weaned off pregabalin with residual but tolerable mild numbness and aching of the feet.

Past medical history was significant for hypertension treated with amlodipine 10 mg daily.

We will list the relevant clinical findings on examination. Gait was steady with normal tandem-walking. Heel and toe-walking were normal. Cranial nerve examination was normal with normal trigeminal nerve sensory testing to touch in all three trigeminal divisions bilaterally.

Power testing of the arms and legs was normal using medical research council (MRC) grading. Cerebellar examination revealed normal heel-to-shin and finger-to-nose testing. Romberg sign was absent with absence of pseudoathetosis of the fingers with the outstretched arms and eyes closed. Deep tendon reflexes were normal except for trace ankle jerks bilaterally. Sensory examination of the feet revealed mild diminished pin-prick testing of the toes with absent vibratory testing of the toes bilaterally. The findings on examination are consistent with a low-grade sensory neuropathy.

**Figure 1:**
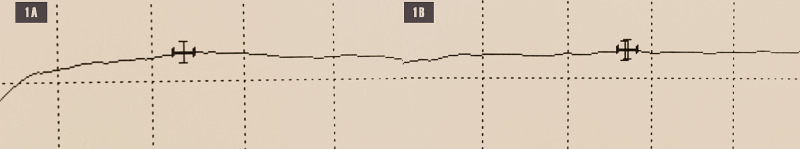
Sensory nerve action potentials. 1A - absent right sural sensory amplitude; 1B - absent right superficial peroneal sensory amplitude Nerve Conduction Study

A nerve conduction study (NCS) of the feet revealed absent sural and peroneal sensory nerve action potentials bilaterally. Peroneal and tibial motor amplitudes and velocities were normal in both feet. This is consistent with sensory neuropathy (Figure 1).

**Figure 2:**
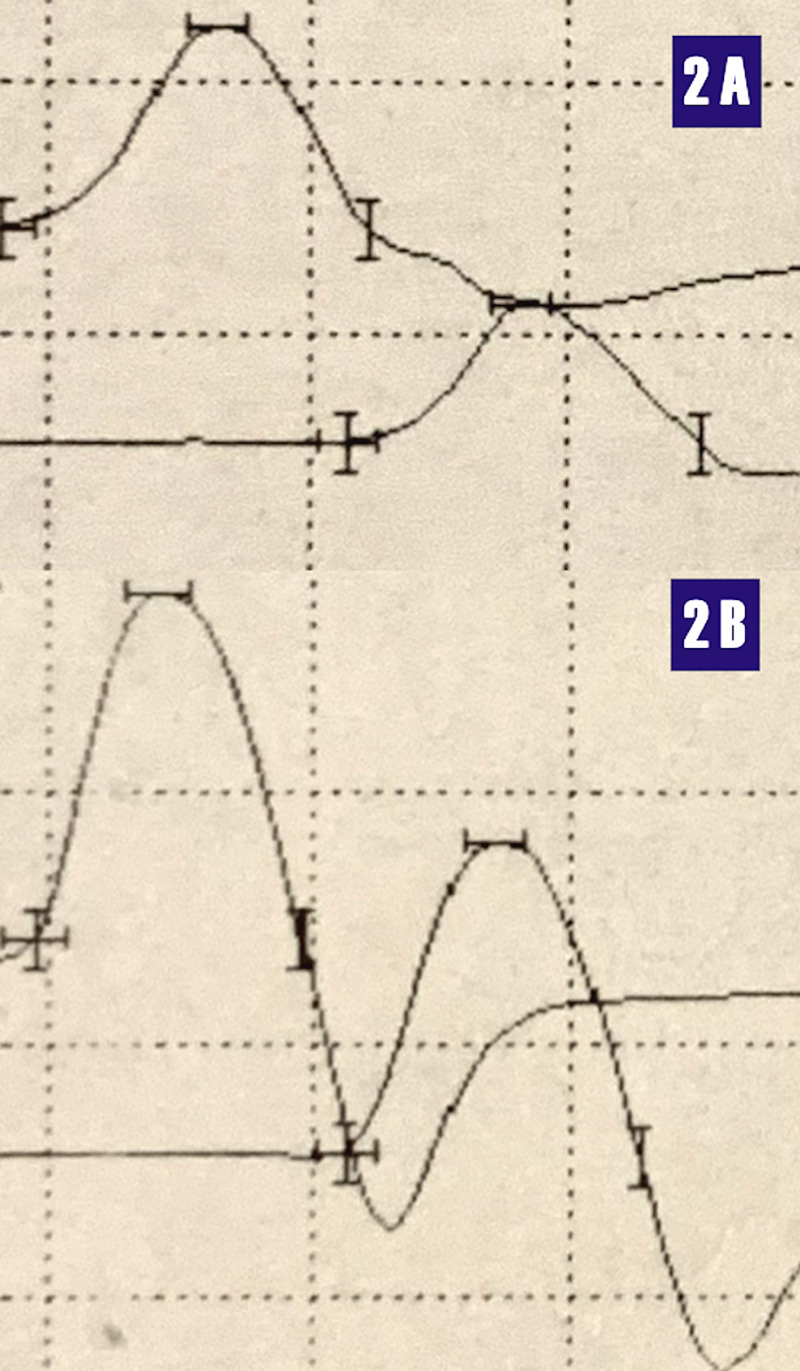
Motor compound muscle action potentials. 2A - right peroneal motor nerve; 2B - right tibial motor nerve Nerve Conduction Study

The NCS preserved motor findings are displayed in Figure 2. 

Our case conforms to the typical profile of a thalidomide-induced polyneuropathy; a cumulative dose of at least 20 grams, predominantly sensory neuropathy and the absence of power loss or a frank sensory ataxia. The latter findings, if present, should prompt the search for an alternate diagnosis. Furthermore, as expected, the discontinuation of drug therapy was associated with a gradual improvement of symptoms. 

Due to the recent resurgence of the use of thalidomide, we were propelled to present the typical case and outline the clinical profile and red flags of presentation. Familiarity with the neurotoxicity of this agent should be highlighted.

## Discussion

**Figure 3:**
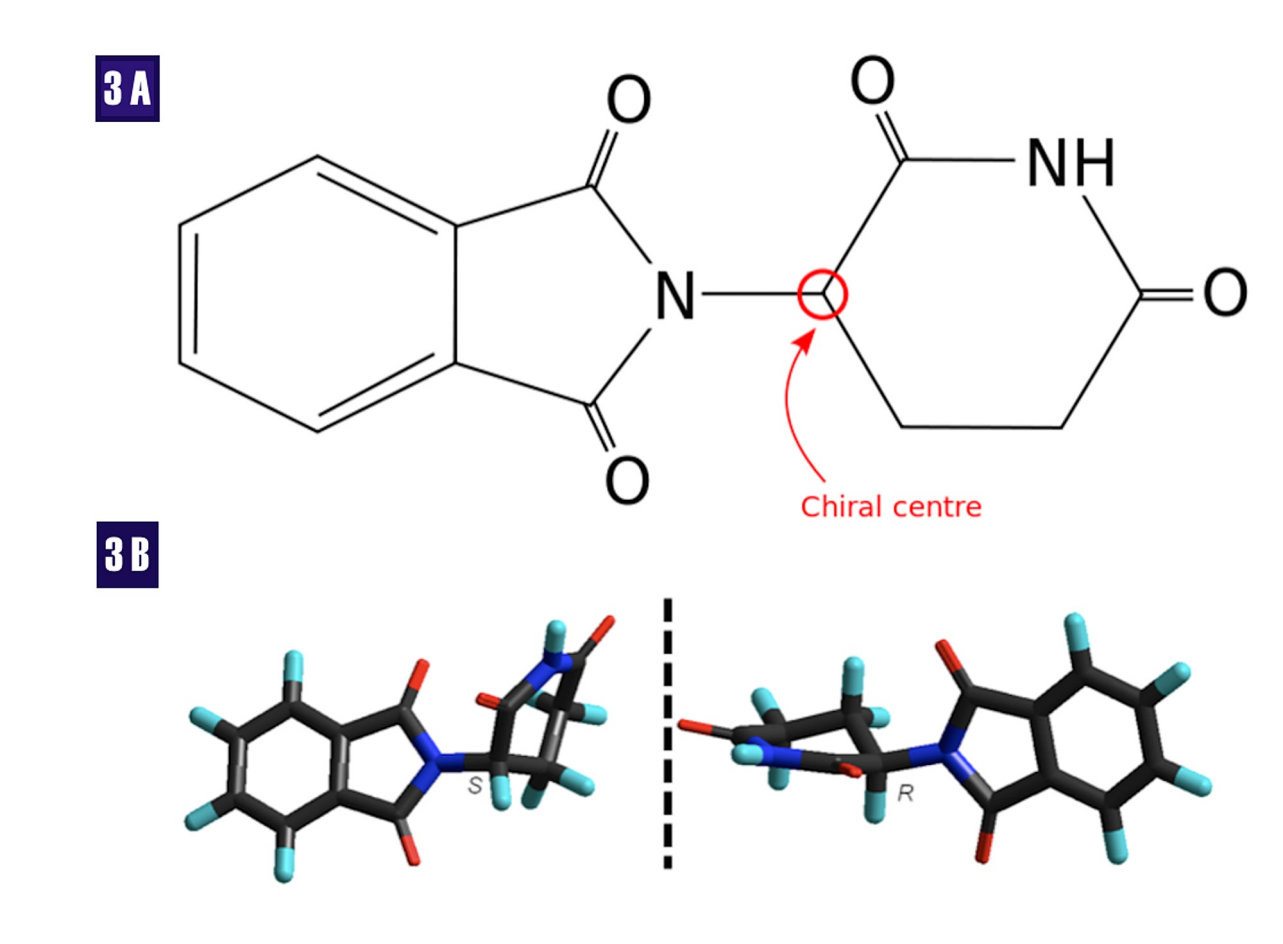
Thalidomide molecule. 3A - Thalidomide rings resemble the ring of the nucleotide guanine; 3B - The S- and R- stereoisomers Sinister (S), rectus (R), oxygen (O), nitrogen (N), hydrogen (H)

Thalidomide exists as a racemic mixture of two enantiomers that exist as mirror images of each other. These images are non-superimposable, a phenomenon known in physics as parity. This stereochemistry (geometry in physics) gives them optical properties whereby the plane of polarized light is rotated clockwise (+) or dextrorotatory (D), also referred to as rectus (R) and counterclockwise (-) or levorotatory, also known as sinister (S). The chiral center, the part of the molecule that imparts handedness, is usually a carbon molecule (Figure 3). 

The bewildering panorama of the molecular biology of thalidomide and its anti-inflammatory, immunomodulatory, and anti-angiogenesis effects, and its subsequent effects on embryogenesis and neuronal functions are listed in Table 1. 

**Table 1:** The molecular biology of the thalidomide molecule and its subsequent pathology Tumor necrosis factor (TNF), messenger ribonucleic acid (mRNA), fibroblast growth factor (FGF), interleukin (IL), vascular endothelial growth factor (VEGF), polyneuropathy, organomegaly, endocrinopathy, monoclonal gammopathy and skin changes (POEMS), thymic helper cells (Th), insulin growth factor (IGF), deoxyribonucleic acid (DNA), nuclear factor (NF)

Study	Molecular biology	Effects
Amato RJ, et al. [7]	reduces TNF-alpha by accelerated degeneration of mRNA	reduces inflammation in TNF-alpha immune mediated diseases
George A, et al. [8]	recruits epineural IL-10 macrophages and increases dorsal horn met-enkephalins	may reduce neuropathic pain
Kuwabara S, et al. [9]	reduces serum VEGF and inhibits angiogenesis	clinical and electrophysiological improvement in POEMS
Park SJ, et al. [10]	inhibits neointimal hyperplasia	reduced FGF and TNF-alfa
McHugh SM, et al. [11]	shift from pro-inflammatory Th1 to anti-inflammatory Th2 cytokines	broad-spectrum anti-inflammatory action
Mori T, et al. [12]	inhibits cereblon - E3 ubiquitine ligase	embryopathy
Stephens TD, et al. [13]	inhibit binding of IGF-1 and IGF-2 to alpha and beta integrins	reduce angiogenesis and bud outgrowth.
Parman T, et al. [14]	oxidizes DNA	thalidomide molecule similar to guanine nucleotide
Yasui K, et al. [15]	inhibits NF-kappa	inhibit granulocyte- mediated tissue injury

How does thalidomide induce its toxic effects on neurons? It may lead to dorsal root ganglion toxicity or a length-dependent axonopathy [6]. Inhibition of ubiquitination and reduced clearance of proteins [12] and oxidation of DNA leading to Wallerian degeneration is speculative at this point [14]. There is a need for electron-microscopic studies of sural nerve biopsies of afflicted patients to look for accumulation of deposits (toxic axonopathy) such as axonal spheroids (accumulation of cytoskeletal proteins) and abnormal mitochondria (oxidative phosphorylation abnormalities) or abnormal granular deposits in the endoplasmic reticulum.

The pleomorphic molecular mechanisms of the thalidomide molecule explain its wide-ranging therapeutic effects. From the neurological therapeutic point of view, thalidomide has benefits for two diseases: polyneuropathy, organomegaly, endocrinopathy, monoclonal gammopathy and skin changes (POEMS syndrome) and advanced glioblastoma.

The POEMS syndrome is an osteosclerotic myeloma associated with a monoclonal spike of immunoglobulin IgA or IgG and a lambda-chain paraproteinemia. POEMS is an aggressive demyelinating sensory-motor polyneuropathy associated with bilateral lower and upper limb weakness and difficulty walking by two years. One-third of patients have papilledema and testicular atrophy and gynecomastia are not infrequent. POEMS can mimic chronic inflammatory demyelinating polyneuropathy (CIDP) and treatment-resistant CIDP should raise suspicion for POEMS. Treatment for POEMS can include radiotherapy for osteosclerotic lesions, alkylating agents such as cyclophosphamide, and bone marrow transplant. Elevated serum levels of vascular endothelial growth factor (VEGF) are seen in most patients and this has prompted the use of thalidomide in POEMS. Kuwabara et al. treated nine patients who were not candidates for alkylating agents or bone marrow transplant with thalidomide. All patients had elevated serum VEGF. Treatment with thalidomide led to clinical improvements, reduced serum VEGF levels, and improvement in electrophysiological parameters in all patients [8].

The anti-angiogenesis and sedative properties of thalidomide have also been used to treat advanced gliomas. The benefits include prolonged survival and better sleep hygiene [3].

The polyneuropathy of thalidomide is mostly a sensory neuropathy associated with a large fiber neuropathy (numbness, mild unsteadiness) and/or a small fiber neuropathy (tingling, burning pain, aching, shooting pain). The development of a polyneuropathy is related to the duration of treatment and the cumulative dose of drug, with doses exceeding 20 grams increasing the risk to 50%. The polyneuropathy is mostly axonal, rarely demyelinating and is usually reversible with drug discontinuation. Clinically, the ankle-deep tendon reflexes are normal, diminished, or absent. There is usually a length-dependent polyneuropathy to pin-prick. Vibratory sense may be diminished but joint-position sense at the toes is usually preserved. The Romberg sign is usually absent (normal proprioception) and the patient is almost always able to stand on his or her heels (absence of weakness) (Table 2).

**Table 2:** Thalidomide treated patients - emphasis on predominantly sensory neuropathy and dose-dependent effects on development of a neuropathy Somatosensory evoked potential (SSEP), dorsal root ganglion (DRG), grams (g)

Study	Number of patients treated with thalidomide	Sensory findings	Sensory-motor findings	Noteworthy findings
Lagueny A, et al. [16]	13	13	0	Two patients with prolonged SSEP's - ? dorsal column / DRG involvement
Briana C, et al. [17]	14	7	1	No correlation between cumulative dose of thalidomide and emergence of neuropathy
Cavaletti G, at al. [18]	65	46	0	A cumulative dose greater than 20 g is related to development of a neuropathy
Chaudhry V, et al. [19]	7	7	3	Dose-dependent effect. Sural nerve biopsy demonstrating Wallerian degeneration and loss of myelinated fibers
Bastuji-Garin S, et al. [20]	135	34	no data provided	No neuropathy for daily doses of less than 25 g daily. The risk increased with increased daily dosing

If the patient develops a sensory ataxia, Romberg sign, inability to stand on the heels or tip-toes, diffuse numbness (truncal, facial or appendicular), cranial nerve palsy, or an autonomic neuropathy (orthostatic intolerance, dry eyes, dry mouth, urogenital dysfunction) or asymmetric or multi-focal weakness, then this should raise a red flag and an alternate etiology pursued. An alternate etiology may include a hereditary sensory-motor polyneuropathy, other inflammatory neuropathy such as CIDP or a paraneoplastic ganglioneuropathy. 

## Conclusions

A thalidomide-induced polyneuropathy is mostly a sensory neuropathy. It is a cumulative dose-dependent polyneuropathy that is usually reversible with discontinuation. We listed " red flag " unusual presentations such as difficulty standing on the heels or sensory ataxia, which should prompt a search for an alternate diagnosis. The stereo-chemistry and molecular mechanisms of the thalidomide molecule are manifold and worthy of review as the indications for its clinical applications has exploded over the last two decades. 
